# Computational methods for alternative polyadenylation and splicing in post-transcriptional gene regulation

**DOI:** 10.1038/s12276-025-01496-z

**Published:** 2025-08-14

**Authors:** Naima Ahmed Fahmi, Sourav Saha, Qianqian Song, Qian Lou, Jeongsik Yong, Wei Zhang

**Affiliations:** 1 Department of Computer Science, University of Central Florida, Orlando, FL USA; 2 Department of Health Outcomes and Biomedical Informatics, University of Florida, Gainesville, FL USA; 3 Department of Biochemistry, Molecular Biology and Biophysics, University of Minnesota Twin Cities, Minneapolis, MN USA

**Keywords:** Computational models, Post-translational modifications

## Abstract

Alternative polyadenylation (APA) and alternative splicing (AS) are essential post-transcriptional mechanisms that enhance transcriptome diversity and regulate gene expression across various biological contexts. APA modifies transcript stability, localization and translation efficiency by generating mRNA isoforms with distinct 3′ untranslated regions or coding sequences, while AS alters protein diversity through exon inclusion or exclusion. The advent of high-throughput RNA sequencing has driven the development of computational methods to systematically identify, quantify and analyze APA and AS events, shedding light on their regulatory roles in normal physiology and disease. These methods can be broadly categorized based on their underlying methodologies and the data types they process, with specialized tools designed for both bulk and single-cell RNA sequencing. Here, in this Review, we provide a comprehensive overview of computational strategies for APA and AS detection and differential analysis, highlighting their advantages, limitations and applications. In addition, we explore techniques specifically tailored for single-cell RNA sequencing. We enhance our understanding of APA and AS regulation across diverse biological systems by summarizing recent advancements, offering new insights into gene regulation at both the population and single-cell levels.

## Exploring post-transcriptional regulation via alternative polyadenylation and splicing

This article explores how genes in complex organisms, such as humans, generate different protein isoforms through post-transcriptional mechanisms such as alternative polyadenylation and alternative splicing. The authors discuss the complexity of these regulatory processes and how they are analyzed using high-throughput RNA sequencing technologies. The study reviews various computational methods developed to detect alternative polyadenylation and alternative splicing events from RNA sequencing data. These methods help identify alternative transcript isoforms by detecting variable polyadenylation sites and splicing patterns in various conditions. The research highlights the importance of these methods in understanding gene regulation and their potential role in diseases such as cancer. While considerable progress has been made, challenges remain due to sequencing biases and technical limitations. They suggest future research should focus on integrating new technologies and data types to improve our understanding of post-transcriptional gene regulation.

This summary was initially drafted using artificial intelligence, then revised and fact-checked by the author.

## Introduction

Most eukaryotic genes consist of multiple exons and introns, with introns being spliced out to produce mature mRNAs^1^. During their processing, eukaryotic mRNAs also acquire polyadenylated tails^2^. These processes occur cotranscriptionally or post-transcriptionally, and alterations via alternative polyadenylation (APA) and alternative splicing (AS) can generate multiple isoforms from a single gene^3,4^.

**Fig. 1:**
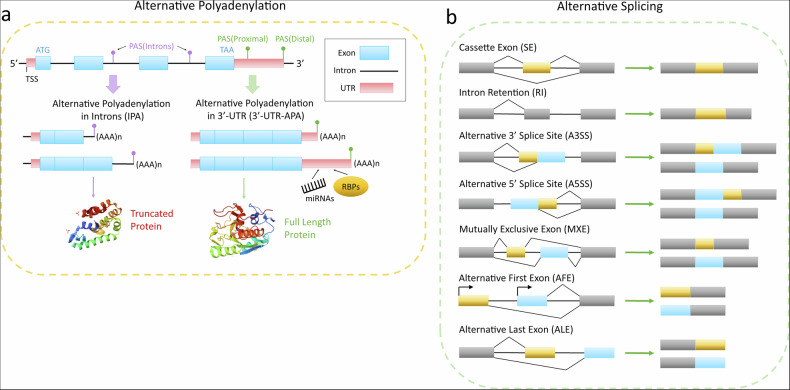
Different forms of eukaryotic APA and AS events. **a** The two types of APA, that is, IPA and UTR-APA, and their impact on the functional proteome. PAS, polyadenylation site; RBP, RNA-binding protein; TSS, transcription start site. **b** A schematic representation of the seven major types of AS.

There are two types of APA, each influencing mRNA length in the transcriptome and impacting the functional proteome differently, as illustrated in Fig. 1a. APA in the last exon generates mRNAs with truncated 3′-UTR APA (UTR-APA), promoting protein synthesis because the 3′-UTR acts as a binding platform for microRNAs and RNA-binding proteins, serving as a regulatory scaffold for gene expression^5–7^. On the other hand, intronic APA (IPA), which occurs within a gene’s intron, results in mRNA truncation within the coding region. This often leads to the production of truncated proteins that lack or replace functional or regulatory domains in their C-terminus^8^. Isoforms derived from IPA have been shown to function uniquely within cells, with the potential to strongly influence biological processes by overriding regulatory pathways^9–11^.

Similar to IPA, AS in the coding region critically influences nearly all aspects of protein functionality by altering their functional domains^5^. The seven major types of AS event are illustrated in Fig. 1b. Numerous studies have highlighted tissue-specific AS events in genes and their associated tissue-specific functions^12–14^. Notably, more than 95% of pre-mRNAs with multiple exons in humans undergo AS, producing over 250,000 protein isoforms, despite the human genome containing only ~25,000 genes^15–17^. Moreover, approximately 50% (~12,500 genes) of annotated human genes harbor at least one IPA event^18,19^. These regulatory mechanisms allow cells to generate diverse protein isoforms from a single gene, expanding the genome’s functional capacity and enabling multifaceted biological roles.

Over the past few decades, the rapid development of next-generation sequencing technologies, particularly RNA sequencing (RNA-seq)^20^ and single-cell RNA-seq (scRNA-seq)^21^, has revolutionized the ability to detect transcriptome-wide APA and AS events. These technologies have enabled high-resolution profiling of transcriptomes, uncovering the complexity of RNA processing at both bulk and single-cell levels. In the last 15 years, researchers have developed tens of methods and bioinformatics pipelines specifically designed to identify these events. These approaches allow for the detection of both annotated events, which are already documented in genomic databases, and unannotated (that is, de novo) events that reveal novel transcript variants. Furthermore, the advent of scRNA-seq has expanded this capability to single-cell resolution^21^, offering a detailed view of APA and AS dynamics across diverse cell types and conditions. This progress has substantially enhanced our understanding of RNA regulation and its role in cellular, tissue-specific and cell-type-specific functions.

This review provides a comprehensive overview of computational methods for detecting post-transcriptional regulation events using RNA-seq and scRNA-seq data, with a focus on APA and AS. We categorize and compare available methods across different analytical frameworks, highlighting their advantages and limitations. Moreover, we discuss key resources and tools to assist researchers, including molecular biologists, in selecting the most suitable approaches to address specific challenges in APA and AS analysis.

## Alternative Polyadenylation

Methods for analyzing APA typically involve two distinct steps: polyadenylation site (poly(A) site) prediction or identification and differential APA event detection. The first step focuses on detecting poly(A) sites, either by utilizing annotated databases or by applying computational models trained on annotated sites or sequencing data, such as RNA-seq or 3′-end sequencing (3′-end-seq), to identify novel or actively expressed sites. The second step involves identifying differential APA events by comparing poly(A) site usage across different biological conditions, uncovering changes in gene regulation, transcript isoform expression or cellular responses. Various statistical and machine learning algorithms have been developed to enhance the accuracy and reliability of differential APA detection. Here, we will first describe existing annotated poly(A) site resources (‘Annotated poly(A) sites’ section), followed by computational methods specifically for poly(A) site prediction (‘Computational methods for poly(A) sites prediction only’ section). Next, we will discuss tools focused solely on detecting differential APA events (‘Computational methods for differential APA events detection only’ section) and those designed for comprehensive APA analysis encompassing both steps (‘Computational methods for APA analysis’ section). Lastly, we will review recent advancements in APA analysis at the single-cell level (‘APA analysis at single-cell level’ section). A summary of computational methods for APA analysis is presented in Tables 1 and 2.

**Table 1:** Computational methods for APA analysis.

Name	APA location	Approach	Language	Novel APA	Differential APA	Year
3′T-fill^26^	UTR-APA	New experimental technique	Existing packages	Yes	No	2013
Naive Bayes classifier^28^	UTR-APA	poly(A) sites prediction (ML)	Perl	Yes	No	2013
change-point^34^	UTR-APA	Detect changes in read density based on annotated poly(A) sites	Java	No	Yes	2014
DaPars^39^	UTR-APA	Modeling read density changes	Python	Yes	Yes	2014
GETUTR^27^	UTR-APA	Modeling read density changes	Python	Yes	No	2015
IsoSCM^40^	UTR-APA	Modeling read density changes	Java	Yes	Yes	2015
Roar^35^	UTR-APA	Detect changes in read density based on annotated poly(A) sites	R	No	Yes	2016
Omni-PolyA^29^	Poly(A) site identification	poly(A) sites prediction (ML)	Webtool	Yes	No	2017
QAPA^36^	UTR-APA	Detect changes in read density based on annotated poly(A) sites	Python, R	No	Yes	2018
PAQR_KAPAC^37^	UTR-APA	Detect changes in read density based on annotated poly(A) sites	Python, R	No	Yes	2018
APAtrap^41^	UTR-APA	Detect changes in read density	R, Perl	Yes	Yes	2018
IntMAP^42^	UTR-APA	Modeling read density changes	MATLAB	Yes	Yes	2018
TAPAS^43^	UTR-APA, IPA	Detect changes in read density	Shell, R	Yes	Yes	2018
APARENT^30^	Poly(A) site identification	poly(A) sites prediction (DL)	Python	Yes	No	2019
DeeReCT-PolyA^32^	Poly(A) site identification	poly(A) sites prediction (DL)	Python	Yes	No	2019
mountainClimber^110^	UTR-APA, IPA	Detect changes in read density	Python	Yes	Yes	2019
SANPolyA^31^	Poly(A) site identification	poly(A) sites prediction (DL)	Python	Yes	No	2020
APAlyzer^38^	UTR-APA, IPA	Detect changes in read density based on annotated poly(A) sites	R	No	Yes	2020
PolyA-Miner^46^	UTR-APA	Modeling peak changes with 3′-end-seq	Python	Yes	Yes	2020
Aptardi^44^	UTR-APA	poly(A) sites prediction (DL)	Shell, Python	Yes	Yes	2021
MAAPER^111^	UTR-APA, IPA	Modeling read density changes	R	Yes	Yes	2021
IPAFinder^47^	IPA	Modeling read density changes	Python	Yes	Yes	2021
DeeRect-APA^33^	Poly(A) site identification	poly(A) sites prediction (DL)	Python	Yes	No	2022
APA-Scan^112^	UTR-APA	Detect changes in read density	Python	Yes	Yes	2022
APAIQ^113^	UTR-APA, IPA	poly(A) sites prediction (DL)	Python	Yes	Yes	2023
InPACT^48^	IPA	poly(A) sites prediction (DL)	Python	Yes	Yes	2024
PolyAMiner-Bulk^49^	UTR-APA	poly(A) sites prediction (DL)	Python	Yes	Yes	2024

‘Language’ indicates the programming language used to implement the pipeline. ‘Novel APA’ indicates whether the method can detect novel (unannotated) APA events. ‘Differential APA’ specifies whether the method supports differential APA analysis between conditions. ‘Year’ denotes the publication year of the original manuscript. ML, machine learning method; DL, deep learning method.

**Table 2:** Computational methods for single-cell APA analysis.

Name	APA location	Approach	Language	Novel APA	Differential APA	Year
scAPA^50^	UTR-APA, IPA	Peak calling-based method	R	Yes	Yes	2019
Sierra^51^	UTR-APA, IPA	Peak calling-based method	R	Yes	Yes	2020
scAPAtrap^52^	UTR-APA, IPA	Peak calling-based method	R	Yes	Yes	2021
scDAPA^56^	UTR-APA	Detect changes in read density	Shell, R	Yes	Yes	2020
SCAPTURE^53^	UTR-APA, IPA	Peak calling-based method (DL)	Shell, R, Python	Yes	Yes	2021
SAPAS^114^	UTR-APA, IPA	Peak calling-based method	Python, R	Yes	Yes	2021
scDaPars^54^	UTR-APA	Modeling read density changes	R	Yes	Yes	2021
SCAPE^55^	UTR-APA	Peak calling-based method	Python, R	Yes	Yes	2022

‘Language’ indicates the language used to implement the pipeline. ‘Novel APA’ indicates whether the method can detect novel (unannotated) APA events. ‘Differential APA’ specifies whether the method supports differential APA analysis between cell types or identifies cell-type-specific APA events. ‘Year’ denotes the publication year of the original manuscript. DL, deep learning method.

### Annotated poly(A) sites

poly(A) sites play a crucial role in post-transcriptional gene regulation, and several databases have been developed to catalog and analyze them. Most APA analysis methods for analyzing APA rely on annotated poly(A) sites either to train models for detecting novel or expressed sites or to identify differential APA events across biological conditions. PolyA_DB^22^ is a comprehensive resource that compiles experimentally identified poly(A) sites across multiple species, integrating data from expressed sequence tags and full-length complementary DNA sequences. PolyASite^23^ focuses on poly(A) sites in humans, mice and worms, leveraging high-throughput 3′-end-seq data for precise mapping of poly(A) sites. APADB^24^ extends poly(A) site annotation to include functional insights, such as tissue-specific APA patterns and their regulatory mechanisms. In addition, APASdb^25^ offers a platform for studying APA events, utilizing RNA-seq data to explore their roles in gene expression regulation. These databases provide valuable resources for understanding polyadenylation dynamics and serve as critical evidence for detecting novel poly(A) sites.

### Computational methods for poly(A) sites prediction only

Several methods focus exclusively on poly(A) site prediction, which can generally be categorized into two main groups based on their approach. The first group consists of early methods that rely on RNA-seq read coverage or RNA-seq signals to detect expressed poly(A) sites. Examples include 3′T-fill^26^ and GETUTR^27^, which identify poly(A) sites directly from RNA-seq data by analyzing transcript-end signals. The second group focuses on training classification models to predict novel poly(A) sites using annotated poly(A) sites or 3′-end-seq data as training references. These models utilize input features such as the surrounding nucleotide sequences (that is, DNA sequences) and known motifs associated with poly(A) sites. These classification models range from traditional machine learning approaches to advanced deep learning-based architectures. For instance, canonical models such as the naive Bayes classifier^28^ and random forests^29^ have been applied in this context. In recent years, deep learning models have shown notable progress. APARENT^30^ uses a one-hot encoded DNA sequence as input, with two convolutional layers and a dense layer for poly(A) site prediction. SANPolyA^31^ employs a self-attention mechanism to capture patterns for poly(A) site identification. DeeReCT-PolyA^32^, a convolutional neural network (CNN)-based model, is designed to recognize 12 different human poly(A) signals (for example, ATTAAA and AATAAA) for site prediction. Moreover, the same research group developed DeeReCT-APA^33^, a CNN-long short-term memory model, to estimate the usage levels of all poly(A) sites for a gene, leveraging 3′-end-seq data for this purpose. Notably, most methods in the second group do not require RNA-seq data for poly(A) site prediction, making them versatile for applications where only genomic or annotated data are available.

### Computational methods for differential APA events detection only

Several methods rely on annotated poly(A) sites and focus on detecting differential APA events between different biological conditions. These methods bypass the need for de novo site identification, instead using statistical or computational frameworks to compare poly(A) site usage. change-point^34^ employs a change-point model with a likelihood ratio test to detect shifts in RNA-seq read density along transcripts, highlighting differential poly(A) site usage. Roar^35^ evaluates shifts in poly(A) site selection by contrasting RNA-seq coverage over regions proximal and distal to poly(A) sites across conditions, applying Fisher’s exact test to detect differential events. Both change-point and Roar assume the presence of only two poly(A) sites within the 3′ untranslated regions (3′-UTR) region. QAPA^36^ quantifies APA by calculating the proportion of transcript isoforms ending at each annotated poly(A) site, using RNA-seq data to estimate site usage across samples and conditions. KAPAC^37^ identifies *k*-mers that influence poly(A) site choice by modeling poly(A) site usage as a linear function of *k*-mer occurrences within terminal exons. Its companion tool, PAQR, segments 3′-UTRs at annotated poly(A) sites by iteratively minimizing squared deviations in RNA-seq read coverage to detect differential poly(A) site usage. APAlyzer^38^, an R package, performs differential APA analysis for both 3′-UTR and intronic regions, employing a Fisher’s exact test and *t*-test to identify significant events.

### Computational methods for APA analysis

Most computational methods for APA analysis are designed to perform both steps: detecting novel poly(A) sites and identifying differential APA events. Below, we introduce several representative methods, with additional methods summarized in Table 1. DaPars^39^ uses a regression-based model to infer proximal poly(A) sites and calculates the Percentage of Distal PolyA Usage Index (PDUI) to quantify APA dynamics between conditions. This approach is effective for detecting significant APA events, such as 3′-UTR shortening or lengthening, and integrates APA changes with clinical data for survival analysis. IsoSCM^40^ employs a Bayesian change-point inference algorithm to annotate tandem 3′-UTR isoforms from RNA-seq data. By segmenting RNA-seq read density profiles into regions of distinct 3′-UTR lengths, IsoSCM enables the discovery of novel APA events without requiring prior annotation. APAtrap^41^ combines a sliding window-based approach with a statistical framework to refine 3′-UTR annotations, detect poly(A) sites and identify differential APA usage across samples. A mean squared error (MSE) model minimizes the difference between observed and expected RNA-seq read coverage to precisely locate poly(A) sites. The method uses the percentage difference index to quantify differences in poly(A) site usage between two samples. IntMAP^42^ integrates RNA-seq and 3′-end-seq data to identify expressed poly(A) sites and quantify poly(A) usage. A chi-squared test is applied to detect significant differential APA events between conditions. TAPAS^43^ utilizes the Pruned Exact Linear Time algorithm to detect potential poly(A) sites by identifying sharp changes in RNA-seq read coverage. Differentially expressed poly(A) sites between biological conditions are identified using a negative binomial model. Aptardi^44^ applies a bidirectional long short-term memory (biLSTM) recurrent neural network to 27 features derived from DNA sequence and RNA-seq data to predict poly(A) sites. DESeq2^45^ is then applied to identify differential APA events between biological conditions. PolyA-Miner^46^ is specifically designed for 3′-seq data and uses vector projections and non-negative matrix factorization to detect and assess differential APA events. Peaks filtered from 3′-seq data are considered as potential poly(A) sites for further analysis.

Unlike the methods above, IPAFinder^47^ and InPACT^48^ focus specifically on IPA identification. IPAFinder calculates the MSE of read coverage for upstream and downstream segments of intronic regions split by potential breakpoints. The ratio of (MSE upstream + MSE downstream) to the MSE of the entire intron, termed RatioMSE, is used to identify intronic poly(A) sites. DEXSeq is then applied to detect differential IPA events. InPACT integrates a CNN with two convolutional-pooling layers and one fully connected layer to scan intronic regions for candidate poly(A) sites. A random forest classifier validates these sites using features derived from RNA-seq alignments. InPACT quantifies intronic poly(A) isoforms using Salmon and detects differential IPA events with a Dirichlet-multinomial distribution model (via DRIMSeq).

The group that developed PolyA-Miner also introduced the first BERT language model, PolyAMiner-Bulk^49^, to identify poly(A) sites and analyze APA. PolyAMiner-Bulk employs an attention-based deep learning model, C/PAS-BERT, to predict poly(A) sites by leveraging contextual features from nucleotide sequences. For differential APA analysis between biological conditions, the method applies a beta-binomial test, providing a robust statistical framework to detect changes in poly(A) site usage.

### APA analysis at single-cell level

Analyzing APA at the single-cell level requires tailored computational approaches to address data sparsity, variability and transcript complexity. scAPA^50^, Sierra^51^, scAPAtrap^52^ and SCAPTURE^53^ focus on identifying poly(A) sites from scRNA-seq data and quantifying APA dynamics. scAPA and Sierra define 3′-UTR regions and identify peaks from RNA-seq read coverage. scAPA quantifies proximal poly(A) site usage using the Proximal PolyA Usage Index (PUI), while Sierra uses splice-aware peak calling and DEXSeq for differential analysis. scAPAtrap extends peak-based approaches by iteratively refining peaks and anchoring poly(A) sites using A/T stretches. It quantifies APA dynamics using DESeq2 and metrics such as Relative Usage of Distal PolyA Site (RUD). SCAPTURE applies transcript-level peak calling and a deep learning model (DeepPASS) to refine poly(A) site predictions. It generates poly(A) site based annotations and quantifies APA usage with UMI counts.

scDaPars^54^ and SCAPE^55^ rely on statistical or machine learning models for precise APA quantification. scDaPars adapts DaPars’ regression-based model to calculate the PDUI in single cells. It imputes missing APA values using non-negative least squares regression. SCAPE employs a probabilistic mixture model with approximate expectation-maximization to infer poly(A) sites, considering uncertainties in fragment size and poly(A) tail length. It calculates expected poly(A) length to assess APA preferences and supports differential analysis using pseudo-bulk DEXSeq and Bayesian inference.

scDAPA^56^ focuses on detecting cell-type-specific APA differences. It uses a histogram-based method to bin 3′-ends and calculates the site distribution difference index to identify APA changes between cell groups. The results are visualized through smooth density plots and isoform profiles.

These methods provide robust frameworks for detecting APA differences across cell types, enabling comprehensive exploration of transcriptomic complexity in single-cell data.

Overall, the computational methods for APA analysis have markedly improved our understanding of post-transcriptional gene regulation, enabling precise identification of poly(A) sites and their differential usage across biological conditions. By leveraging RNA-seq and 3′-end-seq data, these methods provide insights into transcript isoform expression, cellular responses and disease mechanisms. With ongoing improvements in machine learning, statistical modeling and single-cell technologies, APA analysis continues to evolve, offering powerful tools for studying gene regulation and transcriptome complexity in both bulk and single-cell contexts.

## Alternative Splicing

In general, AS analysis also involves two main steps. First, when transcript annotations are incomplete or unavailable, algorithms reconstruct transcript structures or detect splicing events using RNA-seq data. Second, AS events are quantified using various approaches, including percent spliced-in (PSI) for exon inclusion levels, junction read counts for splice-junction usage, splice graphs for transcript structure inference and isoform quantification to estimate transcript abundances. Differential AS analysis identifies significant changes across conditions (for example, differences in PSI values (dPSI)) using statistical models. Here, we will first describe computational algorithms specifically designed for detecting splicing events by reconstructing transcript structures from RNA-seq data (‘Computational methods for splicing pattern detection on’ section). Following this, we will explore tools dedicated solely to identifying differential AS events (‘Computational methods for identifying differential AS events only’ section) and comprehensive solutions that address both splicing detection and differential analysis (‘Computational methods for AS analysis’ section). Finally, we will review recent computational methods in single-cell AS analysis (‘AS analysis at single-cell level’ section). A comprehensive summary of computational methods used for AS analysis is provided in Tables 3 and 4.

**Table 3:** Computational methods for AS analysis.

Name	Approach	Language	Novel AS	Differential analysis	Visualization	Year
GPSeq^78^	Statistical method	R, C	No	Yes	No	2010
MISO^70^	Statistical method	C, Python	No	Yes	Yes	2010
Solas^77^	Statistical method	R	No	Yes	No	2010
FDM^79^	Graph-based method	Not applicable	Yes	Yes	No	2011
JuncBASE^86^	Junction-centric method	Python	Yes	Yes	No	2011
SpliceTrap^115^	Statistical method	C++, Perl	Yes	No	No	2011
DEXSeq^67^	Statistical method	R	No	Yes	Yes	2012
KIS SPLICE^59^	Graph-based method	C++, Python	Yes	No	No	2012
MATS^82^	Statistical method	C++, Python	Yes	Yes	No	2012
SpliceGrapher^58^	Graph-based method	Python	Yes	No	Yes	2012
Cufflinks^57^	Statistical method	C++, C	Yes	No	No	2012
CuffDiff 2^68^	Statistical method	C++, Shell	No	Yes	No	2013
DiffSplice^80^	Graph-based method	Python	Yes	Yes	Yes	2013
DSGSeq^69^	Statistical method	R, C	No	Yes	No	2013
rDiff^83^	Statistical method	MATLAB, Python	Yes	Yes	No	2013
rMATS^116^	Statistical method	Python, C++	Yes	Yes	No	2014
spliceR^117^	Classification of splicing events	R	No	No	No	2014
MAJIQ^81^	Graph-based method	Python	Yes	Yes	Yes	2016
SGSeq^61^	Graph-based method	R	Yes	No	No	2016
SplAdder^62^	Graph-based method	Python	No	Yes	No	2016
JunctionSeq^84^	Statistical method	R	Yes	Yes	Yes	2016
IRFinder^63^	Detect changes in read density	C++	Yes	No	No	2017
ASGAL^66^	Graph-based method	Python	Yes	No	No	2018
LeafCutter^87^	Junction-centric method	R, Python, C++	Yes	Yes	Yes	2018
SUPPA2^71^	Density-based clustering method	Python	No	Yes	No	2018
JUM^88^	Junction-centric method	Perl, Shell	Yes	Yes	No	2018
CASH^89^	Junction-centric method	Java	Yes	Yes	Yes	2018
Whippet^60^	Graph-based method	Julia	No	Yes	No	2018
IsoformSwitchAnalyzeR^118^	Statistical method	R	No	Yes	No	2019
DARTS^74^	Statistical and deep learning method	Python, R	No	Yes	No	2019
PSI-Sigma^75^	Junction-centric method	Perl	No	Yes	No	2019
BANDITS^73^	Statistical method	R, C++	No	Yes	No	2020
ASpli^85^	Statistical method	R	Yes	Yes	No	2021
Bisbee^90^	Junction-centric method	Python, Matlab,R	Yes	Yes	No	2021
AS-Quant^76^	Detect changes in read density based on transcript annotations	Python	No	Yes	Yes	2021
ScanExitron^64,65^	Junction-centric method	Python	Yes	Yes	No	2021
rMATS-turbo^72^	Graph-based method	Python	Yes	Yes	Yes	2024

‘Language’ indicates the programming language used to implement the pipeline. ‘Novel AS’ indicates whether the method can detect novel (unannotated) AS events. ‘Differential analysis’ specifies whether the method supports differential AS analysis between conditions. ‘Visualization’ indicates whether the pipeline supports visualization of AS events. ‘Year’ denotes the publication year of the original manuscript.

**Table 4:** Computational methods for single-cell AS analysis.

Name	Approach	Language	Novel AS	Differential analysis	Visualization	Year
SingleSplice^91^	Graph-based method	R	Yes	Yes	No	2016
BRIE^93^	Statistical method	Python	No	Yes	No	2017
EXPEDITION^92^	Graph-based method	Python	Yes	Yes	Yes	2017
BRIE2^94^	Statistical method	R	No	Yes	No	2017
CENSUS^95^	Statistical method	Python	No	Yes	No	2021
DESJ-detection^96^	Junction-centric method	R	No	Yes	No	2021
SCASL^97^	Junction-centric method	Python	Yes	Yes	No	2024

‘Language’ indicates the programming language used to implement the pipeline. ‘Novel AS’ indicates whether the method can detect novel (unannotated) AS events. ‘Differential analysis’ specifies whether the method supports differential AS analysis between cell types or identifies cell-type-specific AS events. ‘Visualization’ indicates whether the pipeline supports visualization of AS events. ‘Year’ denotes the publication year of the original manuscript.

### Computational methods for splicing pattern detection only

Methods for detecting splicing patterns often rely on similar graph-based representations (for example, splicing graph) or transcript reconstruction principles. Cufflinks^57^ assembles transcript structures by analyzing splice junctions and read alignments to infer isoforms and quantify their abundances, making it foundational for splicing analysis. Similarly, SpliceGrapher^58^ constructs splice graphs but integrates expressed sequence tags to enhance long-range transcription predictions. KIS SPLICE^59^ and Whippet^60^ also employ graph-based approaches but differ in their focus: KIS SPLICE utilizes compressed De-Bruijn graphs to detect bubble patterns representing AS events, while Whippet leverages contiguous splice graphs for lightweight de novo event identification and quantify them using the expectation-maximization algorithm to compute PSI values.

SGSeq^61^ and SplAdder^62^ are closely related in their use of splice graphs but address different aspects. SGSeq generates graphs from annotations or RNA-seq data and emphasizes local quantification of splicing events by analyzing structurally compatible reads overlapping the start or end of splice variants. SplAdder, integrates RNA-seq data with annotations to augment graphs by adding novel exons and junctions, enabling both discovery and quantification of splicing events, with subsequent differential analysis using tools such as rDiff.

Targeting specific splicing patterns, IRFinder^63^ specializes in quantifying intron retention by calculating intron retention ratios, combining intronic read abundances with spliced read counts to evaluate intron retention levels across samples. ScanExitron^64,65^ identifies exitron splicing events using splice junctions and gene annotations and filters out low-confidence events based on the percent spliced out metric, which quantifies the proportion of transcripts in which a given exitron is spliced. By contrast, ASGAL^66^ applies graph-based alignment to detect a wide range of splicing events, including exon skipping and alternative splice sites, by leveraging RNA-seq reads and refined gap-aware alignment to minimize inconsistencies in annotations.

### Computational methods for identifying differential AS events only

Several methods have been developed to focus solely on differential AS event analysis, relying on changes in read coverage over altered exons to detect events. Methods such as DEXSeq^67^, CuffDiff2^68^, and DSGSeq^69^ leverage statistical models to identify differences in exon usage or isoform expression. DEXSeq employs a generalized linear model (GLM) to compare exon read counts, while DSGSeq uses a gene structure matrix and negative binomial models to relate exons to isoforms. CuffDiff2 extends RNA-seq analysis to isoform-level differences using beta negative binomial modeling, accounting for variability and ambiguous read mappings.

Probabilistic frameworks, including MISO^70^, SUPPA2^71^ and rMATS^72^, focus on quantifying and detecting splicing differences. MISO uses Bayesian inference to assign reads to isoforms, calculating PSI and Bayes factors to assess differential events. Similarly, SUPPA2 computes dPSI values using transcript quantifications and identifies splicing regulatory modules. rMATS combines PSI computation with a likelihood ratio test to evaluate differential splicing across biological conditions.

BANDITS^73^ applies a Dirichlet-multinomial hierarchical Bayesian framework, accounting for transcript lengths and ambiguous read mappings, to perform gene-level and transcript-level testing. DARTS^74^ enhances differential splicing predictions using a deep neural network combined with Bayesian hypothesis testing, leveraging both sequence features and RNA-binding protein levels.

Tools such as PSI-Sigma^75^ and AS-Quant^76^ focus on precise quantification of specific splicing events. PSI-Sigma refines PSI calculations by integrating splice-junction reads, while AS-Quant applies a chi-squared test to evaluate read coverage differences between conditions, providing visualizations for detailed analyses. Solas^77^ integrates multiple statistical frameworks for analyzing alternative exon expression and isoform quantifications, while GPSeq^78^ normalizes RNA-seq data using generalized Poisson models to account for sequencing biases and splicing variation.

Together, these tools provide a robust foundation for analyzing differential AS, each tailored to specific data structures, splicing patterns or experimental conditions.

### Computational methods for AS analysis

Splicing detection and differential AS analysis methods integrate transcript structure inference, splicing event quantification and statistical modeling to identify biologically relevant changes between conditions. These methods can be broadly categorized based on their computational strategies, including graph-based approaches, statistical models and junction-centric algorithms.

Graph-based approaches such as FDM^79^, DiffSplice^80^, MAJIQ^81^ and rMATS-turbo^72^ construct splicing graphs to model transcript structures and detect AS events. FDM reconstructs splicing patterns by building an aligned cumulative transcript graph from RNA-seq data. This graph-based representation models transcript flows through genomic regions, allowing for the detection of exon skipping, intron retention and novel splicing patterns. For differential analysis, FDM is applied as a test statistic to identify genes with significant transcriptional differences between samples. DiffSplice builds transcriptome-wide splice graphs, segmenting them into AS modules that represent diverging paths within a gene’s splicing structure. It quantifies AS events based on read support and employs nonparametric permutation tests to assess statistical significance. MAJIQ models local splicing variations by constructing splice graphs centered around reference exons. It applies Bayesian PSI modeling, bootstrapping and read rate modeling to quantify PSI. For differential AS analysis, MAJIQ estimates dPSI and identifies condition-specific splicing variations. rMATS-turbo extends the weighted splicing graph approach of rMATS, where nodes represent exons and edges represent splice junctions, with a preprocessing step to extract splicing information and a statistical step that detects differential splicing using the same statistical model as rMATS.

Statistical approaches such as MATS^82^, rDiff^83^, JunctionSeq^84^ and ASpli^85^ leverage probabilistic frameworks and count-based models to assess differential splicing. MATS is a Bayesian-based method that detects AS events by modeling exon inclusion levels and applying Markov chain Monte Carlo sampling to compute posterior probabilities of differential AS between conditions. rDiff introduces a statistical framework based on negative binomial models to detect differential AS events. It tests specific isoform regions when annotations are available, whereas for unannotated events, it applies a nonparametric maximum mean discrepancy test to assess differential isoform expression. JunctionSeq extends DESeq2-based statistical models to analyze exon and junction read counts, identifying differential splicing while normalizing data at the gene level to prevent overcounting. It captures both annotated and novel junctions, improving sensitivity. ASpli integrates multiple splicing metrics, including PSI, percent intron retention and percent junction usage, to analyze AS patterns. It employs negative binomial models and GLMs in edgeR for differential splicing detection, providing a comprehensive statistical framework. Although rDiff and JunctionSeq primarily focus on differential AS event prediction, their ability to analyze unannotated events places them within this section.

Junction-centric methods such as JuncBASE^86^, LeafCutter^87^, JUM^88^, CASH^89^ and Bisbee^90^ primarily analyze splice junctions to identify AS patterns. JuncBASE detects and classifies AS events by leveraging splice-junction reads and exon structures. It quantifies isoform expression using read counts and applies statistical tests to determine differential splicing patterns across conditions. LeafCutter clusters introns with shared splice sites to identify AS events. It employs a Dirichlet-multinomial GLM to assess differential intron usage across biological conditions and links genetic variations to splicing changes. JUM quantifies AS events using ‘AS structures’ composed of splice junctions with shared start or end sites. It applies negative binomial models and GLMs to detect splicing differences, categorizing events into conventional and composite AS patterns. CASH reconstructs splice site lists from RNA-seq junction reads and annotates exon–exon junctions. It includes a classification module (SpliceCons) for defining AS event types and a statistical module (SpliceDiff) that applies event-centric and exon-centric differential splicing tests. Bisbee compares splice junctions to known Ensembl exon annotations to predict protein-level effects of AS events. It uses beta-binomial models to detect significant PSI shifts, quantifying differential AS at both the transcript and protein levels.

### AS analysis at single-cell level

Similar to APA analysis at single-cell level, single-cell AS analysis faces several similar challenges, including low read coverage, high dropout rates, biases in transcript capture and limited detection of full-length isoforms due to the sparsity of scRNA-seq data. Unlike bulk RNA-seq, which provides high-depth transcriptomic data, single-cell sequencing often suffers from incomplete transcript representation, making accurate quantification of splicing events difficult. Moreover, technical noise and stochastic gene expression further complicate splicing analysis, requiring specialized computational methods that integrate imputation, statistical modeling and machine learning.

To address these challenges, graph-based methods, such as SingleSplice^91^ and Expedition^92^, reconstruct splice graphs to identify AS modules and predict splicing variations across single cells. SingleSplice uses DiffSplice to segment transcript graphs and applies parametric bootstrapping to differentiate true AS changes from noise. Expedition extends this by incorporating OuTrigger, which detects AS events through de novo splice graph traversal, and Anchor, which assigns splicing modalities using Bayesian inference.

Bayesian and probabilistic approaches such as BRIE^93^, BRIE2^94^ and CENSUS^95^ leverage statistical frameworks to infer missing data and model AS dynamics. BRIE extends MISO’s mixture modeling, applying Bayesian regression with Gaussian priors to model exon inclusion while accounting for dropout effects. BRIE2 improves upon BRIE by integrating cell-type-specific splicing patterns and optimizing computational performance using variational inference, enabling quantitative associations between splicing and cell-level phenotypes. CENSUS, on the other hand, converts TPM values into transcript counts and employs a Dirichlet-multinomial GLM to correct for transcript variability.

Junction-based models such as DESJ-detection^96^ and SCASL^97^ focus on direct splice-junction quantification. DESJ-detection constructs a junction–cell count matrix, filters low-expression junctions using *k*-means clustering and normalizes gene-level expression before applying Limma-trend^98^ for differential AS detection. SCASL extracts junction read counts using LeafCutter, groups splicing events into modules, and applies a two-step imputation method (mean imputation and K-Nearest Neighbors-based iterative refinement) to compensate for missing data. Spectral clustering is then used to classify cells based on their splicing patterns.

Overall, the methods for AS analysis collectively provide a robust framework for detecting AS events and performing differential analysis. The choice of method depends on dataset characteristics, annotation availability and the specific splicing patterns of interest. Similar to APA analysis, AS analysis continues to advance with the increasing availability of RNA-seq and single-cell technologies. These developments enhance the accuracy and resolution of AS event detection and quantification, enabling more precise characterization of transcript diversity across different biological contexts.

## Discussion

Computational methods for analyzing APA and AS have greatly advanced our understanding of transcriptome and post-transcriptional gene regulation. Leveraging high-throughput RNA-seq and scRNA-seq data, these methods apply diverse strategies, including statistical modeling, machine learning and graph-based methods, to detect APA sites, quantify splicing isoforms and assess condition-specific differential usage. These computational approaches not only enable the identification and quantification of transcript isoforms but also provide deeper insights into how transcript variation contributes to gene expression regulation. APA influences mRNA stability, localization and translational efficiency by modulating 3′-UTR length and the inclusion of regulatory elements, while AS alters exon composition, leading to structural and functional changes in protein products^99^. Dysregulation of these processes has been implicated in various diseases, including cancer, neurodegenerative disorders and immune dysfunction^100,101^. Integrative analyses that combine APA and AS profiles with transcriptomic, proteomic and clinical data can uncover functional regulatory programs and disease biomarkers^102^. For example, APA alterations in immune response genes may serve as predictors of therapeutic resistance^103^, while cancer-specific splicing isoforms may highlight novel therapeutic targets^104^. Despite the progress made, several challenges and limitations remain and warrant further critical evaluation.

One major limitation in APA and AS detection lies in the intrinsic biases of RNA-seq and scRNA-seq data. Short-read RNA-seq protocols often exhibit nonuniform read coverage, particularly favoring the 3′ ends of transcripts in polyA-enriched libraries^105^. This bias limits the detection of distal APA sites and leads to inaccuracies when identifying true APA events, especially in genes with multiple closely spaced poly(A) signals. Internal priming, random hexamer artifacts and RNA degradation further complicate interpretation by causing false positives or misleading peaks in read density^106^. Similarly, the detection of AS events is affected by limited read support at exon–exon junctions and the inability of short reads to capture full-length isoforms. These issues become more pronounced in scRNA-seq data, where dropout effects and low sequencing depth per cell reduce sensitivity and increase noise.

While this study has listed numerous methods developed for APA and AS analysis, direct comparative evaluation of their accuracy, computational efficiency, and robustness remains limited in scope. For example, QAPA leverages transcript annotations to quantify 3′-UTR usage but is sensitive to annotation quality and read depth, whereas DaPars performs de novo identification but is more susceptible to false positives in noisy data. In AS analysis, SUPPA enables rapid quantification across large datasets using transcript abundance estimates but may underperform in the presence of complex splicing patterns. By contrast, MAJIQ uses local splicing graphs to detect novel junctions but requires greater computational resources. Despite these differences, few studies provide standardized benchmarks across diverse biological contexts, making it difficult for users to select the most appropriate tool for a given dataset. Future work should emphasize the development of comprehensive benchmarking frameworks that consider runtime, memory usage, event-level accuracy and robustness to read sparsity, especially in single-cell settings.

The advent of large language models, such as DNAbert^107^, Nucleotide Transformer^108^ and HyenaDNA^109^, offers a transformative opportunity for APA and AS analysis. These models, pretrained on genomic sequences, can learn context-dependent regulatory patterns, allowing for improved prediction of poly(A) sites and splicing sites from genomic annotations. By integrating these predictions with RNA-seq data, researchers can evaluate which predicted sites are actively expressed, providing a functional validation framework. This approach not only refines genome annotations but also helps uncover novel regulatory elements that govern transcript diversity. Furthermore, long-read sequencing technologies such as PacBio and Oxford Nanopore can provide direct evidence of APA and AS patterns, reducing the need for computational inference from fragmented short reads. Combining large language models for sequence-based prediction with long-read transcriptomics for experimental validation will enable a hybrid computational-experimental approach, leading to more accurate transcript isoform annotations and a deeper understanding of post-transcriptional regulation in normal and disease contexts. Future research should focus on integrating multimodal data, incorporating chromatin accessibility and RNA-binding protein interactions, to build comprehensive models of gene regulation.
